# Younger and Older Adults’ Cognitive and Physical Functioning in a Virtual Reality Age Manipulation

**DOI:** 10.3389/fragi.2022.851687

**Published:** 2022-04-27

**Authors:** Nils M. Vahle, Martin J. Tomasik

**Affiliations:** ^1^ Department of Psychology and Psychotherapy, University of Witten-Herdecke, Witten, Germany; ^2^ Institute for Educational Evaluation, University of Zurich, Zürich, Switzerland; ^3^ Institute of Education, University of Zurich, Zürich, Switzerland

**Keywords:** virtual reality, aging, age stereotypes, cognitive performance, physical performance

## Abstract

**Objectives:** Age group stereotypes (AGS), especially those targeting old age, affect an individual’s behavior and long-term cognitive and physiological functioning. Conventional paradigms investigating the related mechanisms lack validity and stability. Our novel approach for the activation of self-relevant AGS uses a virtual reality (VR) ageing experience, measuring relevant effects on performance parameters.

**Methods:** In a between-subjects experimental design, young participants embodied either a younger or older avatar in a 3D virtual environment to capture the effects on physical (Study 1; *N* = 68) and cognitive performance (Study 2; *N* = 45). In Study 3 (*N* = 117), the paradigm was applied to older participants.

**Results:** For the younger participants, embodying older avatars was associated with declines in memory and physical performance when compared to the younger avatar age group. Furthermore, the manipulations’ main effects were moderated by negative explicit AGS that matched the respective performance domains. For the older participants, we found no significant performance differences in the two domains investigated.

**Discussion:** The experimental manipulation demonstrated an impact on relevant performance parameters on a motivational and strategic level, especially for strong performance-related AS, but for young participants only. Possible reasons and mechanisms for the differences in younger and older samples’ results are discussed.

## Introduction

Age group stereotypes (AGSs) are widespread (Hummert et al., 1994), and they can be mostly negative when concerning old age (Kotter-Grühn and Hess, 2012). The negative content of these ultimately self-relevant (Levy et al., 2012) stereotypes can have stark consequences for one’s own aging process, particularly in the physical and cognitive domains. In the former, Levy et al. (2002) observed a decline in life expectancy of 50% in people with a negative view on aging. More specifically, holding negative AGS correlates with higher multimorbidity rates (Wurm et al., 2007), poorer cardiovascular health (Levy et al., 2009), and lower physical activity levels (Wurm et al., 2010). Research also showed associations with sensory (Levy et al., 2006) and motor functioning (Robertson and Kenny, 2016). Meanwhile, in the cognitive domain, early studies by Levy and Langer (1994) showed a correlation between AGS and memory performance, and longitudinal associations have been found with working memory (Levy et al., 2012) and general cognitive ability (Uotinen et al., 2005). Furthermore, AGS seems to moderate the association between physical frailty and cognitive functioning (Robertson and Kenny, 2016).

Three mechanisms appear responsible for the association between self-relevant stereotypes and functioning. First, stereotypes might become self-fulfilling prophecies: longitudinal studies show that negative expectations of aging are associated with poorer health behaviors, such as consulting physicians less often, drinking more alcohol, or exercising irregularly (Levy and Myers, 2004). A second explanation is based on findings that people with more negative self-relevant AGS show higher cardiovascular reactivity to stressful stimuli and thus carry a higher cardiovascular morbidity risk (Levy et al., 2000). However, this mechanism alone cannot explain the range of effects found. Finally, stereotype threat, which undermines performance in tasks related to the stereotypes addressed (Steele and Aronson, 1995), has been discussed as a possible mediating process, and this notion was confirmed in a recent meta-analysis by Lamont et al. (2015). The awareness of self-relevant stereotypes posed a stronger threat than seeing information about the actual probabilities of fulfilling them. While we still seek direct methods for reducing the consequences of negative AGS (e.g., Wolff et al., 2014), their presence can be manipulated and intensified in the controlled setting of laboratory experiments.

### Conventional Experimental Activation of Age Stereotypes

Evidence from studies observing a correlation between negative AGS and subsequent declines in performance cannot be considered causal evidence of the phenomenon or of its underlying mechanisms. Experimental studies allowing the deliberate manipulation of stereotypes are therefore needed. For this purpose, several experimental paradigms have been developed to explore competing explanations.

An early approach to activating positive or negative self-relevant AGS used a verbal priming technique, where age-related adjectives (e.g., “confused”) were briefly flashed on a screen, resulting in cognitive activation of the semantic networks related to the concept. Negative AGS priming was shown to cause temporary declines in walking speed (Bargh et al., 1996) and memory performance (Stein et al., 2002); however, these experiments caused controversy regarding their replicability (Rivers and Sherman, 2018), and the approach does not give participants the perspective of an older person; thus, the activation of self-relevant AGS lacks external validity.

Haslam et al. (2012) used two parallel manipulations with age-related self-categorizations by assigning middle-aged participants to either “old” or “young” groups (manipulating self-relevant affiliation with the stereotyped group) and by shaping deficit expectations with newspaper articles on either age-related memory decline or other age-related problems (manipulating the stereotype content). The study found that when participants were labelled “old,” they performed worse on memory tests, particularly when they had been primed with a newspaper article on memory decline. These results support the link between stereotype content and behavioral outcomes. However, the effects were short lived, and the introduction of a social comparison group possibly diluted the self-relevant nature of the stereotype activation, as it added a competitive component.

A more lifelike approach was used by Varkey et al. (2006), where medical students partaking in the *ageing game* were asked to perform simple everyday tasks while wearing technical appliances to reduce their general abilities. With this artificial simulation of living in a frail body, Varkey et al. were able to improve significantly the participants’ empathy and attitudes toward older adult patients. This manipulation offered the continuous embodiment of a physical and perceptual condition commonly associated with older age, even though the design was non-experimental and only allowed an indirect activation of self-related AGS. Still, it demonstrates how complex embodiment manipulations create effective multi-level experiences, leading to promising results.

Eibach et al. (2010) chose an experimental approach with a more complex age-related phenomenology and stronger ecological validity. The manipulation of vision decline and induction of a generation gap experience led to the alteration of subjective age in an adult sample. Furthermore, the successful manipulation of subjective age moderated the influence of AGS on the subjects’ self-evaluation. In a comparable approach, Stephan et al. (2013) presented participants with manipulated performance feedback for a handgrip strength task. Participants in the experimental condition were given positive feedback regarding their performance in comparison to same-aged peers, leading to a reduction in subjective age and an increase in handgrip strength scores for a second measurement. Thus, an experimental manipulation of AGS’ self-relevance is indeed possible by inducing stereotypically old age-related experiences on a perceptual or social level.

Even more complex and visually realistic manipulations are possible whenever stimulus content is created and presented in gaming scenarios in which participants control an avatar whose appearance can be modified. The resulting *Proteus effect* (Yee et al., 2009; named after the shape-changing sea god in Greek mythology) is based on high standardization and multi-sensory stimulus manipulation, implying that self-relevant stereotypes can be activated by visual identity cues, affecting people’s behavior. Earlier studies successfully manipulated social behavior and aggression by increasing body height (Peña et al., 2009) and influenced negotiation interactions by changing the avatar’s attractiveness (Yee et al., 2009). Unfortunately, most studies used a desktop setting, which cannot offer an intuitive immersive experience or multi-level sensory stimulation, and results can only be partially generalized to self-relevant stereotypes.

While the above-mentioned longitudinal relationships offer a view of possible health-related outcome variables, many short-term experimental findings are more difficult to transfer beyond the laboratory and fail to show lasting effects and external validity; hence, proving causality remains complicated. Meanwhile, combining a realistic aging experience with a small-step experimental variation has a continuous impact on perceptions or behaviors and seems the next step necessary to push the limits of our understanding of this psychological mechanism and, furthermore, to develop interventional techniques for the known medical and social implications of strong self-relevant negative AS.

### Using Virtual Reality to Activate Age Group Stereotypes

Together with experts in the field, such as, Slater (2018), Rizzo et al. (2019) or Tuena et al. (2020), we argue that virtual reality (VR) technology enables a realistic immersive experience and hence allows the combination of a high level of standardization and the presentation of true-to-life environments, thus maximizing both internal and external (or ecological) validity. Particularly, users of VR technology can interact with their surroundings more naturally (at least more so than in an interaction with a computer mouse in front of a computer screen), and researchers can manipulate the characteristics of these surroundings in a highly flexible and cost-effective way (at least more flexibly and cost-effectively than setting up, furnishing, or driving to a desired setting). Numerous clinical applications (e.g., Brown et al., 2020) have demonstrated the benefits of an effective immersive experience when simulating general counselling settings (Slater et al., 2019), as well as of applying established therapy approaches to specific conditions, such as obesity and diabetes (Rizzo et al., 2011), eating disorders (Riva et al., 2021), and post-traumatic stress disorder (Rizzo and Shilling, 2017). Earlier studies successfully applied VR to non-clinical topics with regard to interactional or cognitive parameters. Seeing oneself as Sigmund Freud resulted in improved interpersonal problem-solving skills (Osimo et al., 2015), while the virtual embodiment of Albert Einstein improved cognitive performance when compared to a control group (Banakou et al., 2018). Hence, VR scenarios could give participants a realistic aging experience when embodying an older avatar by activating self-relevant AGS in a standardized environment. One implementation of this approach by Reinhard et al. (2020) demonstrated a replication of priming studies using a VR embodiment technique instead of verbal priming; young participants who first embodied an older avatar in a VR scenario showed a decreased walking speed. Furthermore, the approach offers an actual shift in perspective toward an older self, as demonstrated by Sims et al. (2020), who showed age-progressed images to influence young participants’ attitudes toward financial security topics in later life.

Aside from the various applications of VR, some technical details seem to alter the interventional value of the approaches. Slater (2018) argues that VR applications allowing detailed head tracking and the ability to look around at objects provide a higher degree of realism, making it more likely for participants to experience the simulation as if it was really happening. Daher et al. (2017) showed that this even applied to interacting with a virtual agent instead of a real person. If certain technical criteria are met, a strong body ownership illusion is ensured as one possible mechanism behind VR. The body ownership illusion theory (e.g., Kilteni et al., 2015) focuses on the plasticity of the processing of perceptual stimuli. Based on the classic rubber-hand illusion findings (Botvinick and Cohen, 1998), it is argued that VR creates an intersensory bias to perceive the displayed body parts as one’s own (Banakou et al., 2018). The combination of tactile, visual, or auditory stimuli must be coordinated well enough, together with the experience of actually controlling the virtual avatar by moving its virtual head and arms around. By doing so, a realistic body ownership experience is provided, thus activating expectations and stereotypes directly and intuitively that are connected to the virtual avatar. In contrast, the Proteus effect suggests that people in general (Frank and Gilovich, 1988) and computer gamers in particular (Yee et al., 2009) adjust their behavior to their own expectations and stereotypes related to the visual identity cues of their digital avatar. The activation of self-relevant stereotypes takes place indirectly from perceiving one’s own appearance, and it thus depends on appropriate perceptions and semantic interpretations of visual cues.

## Present Studies

**FIGURE 1:**
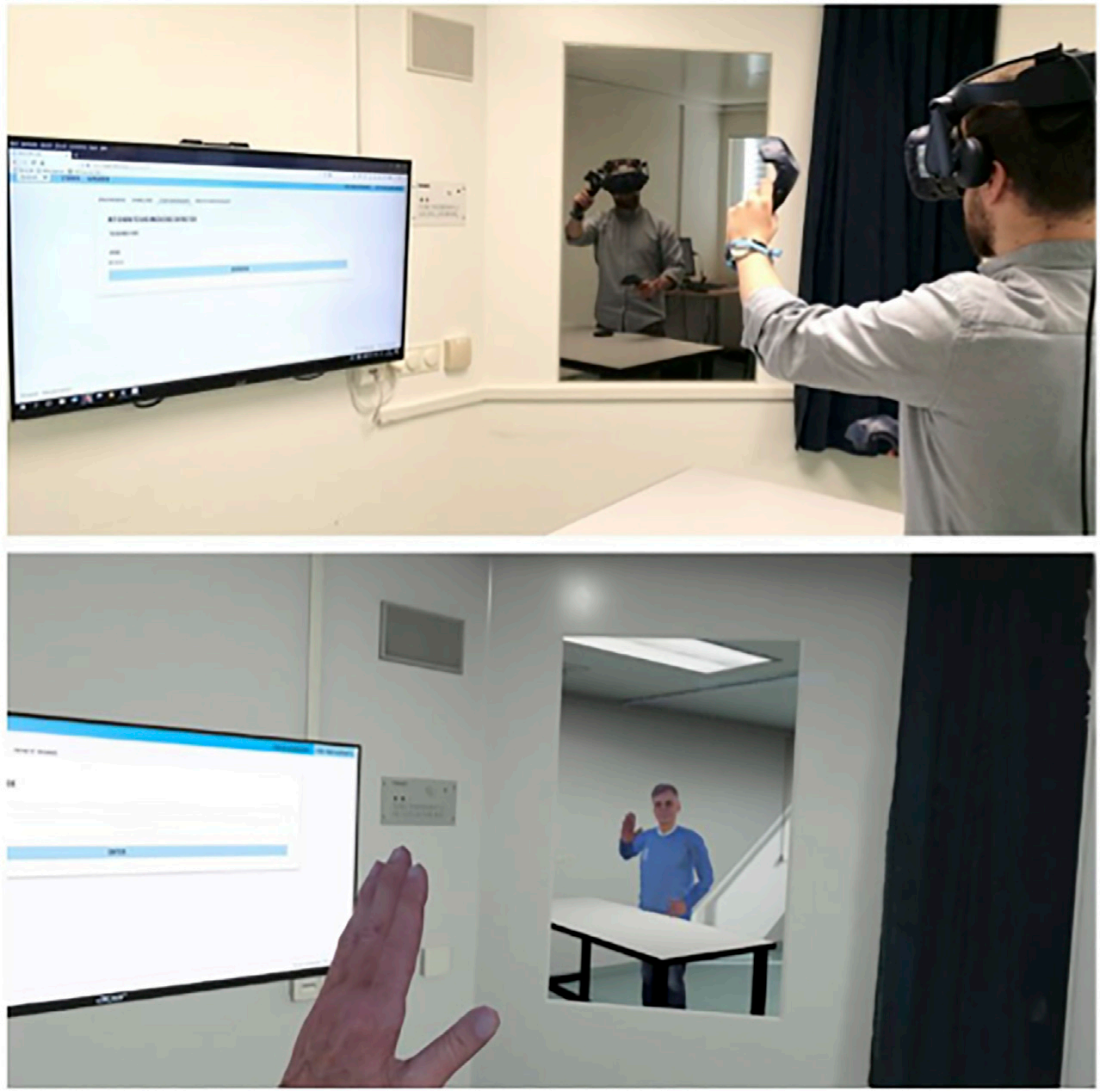
Laboratory room and virtual simulation comparison. Note. See download link below for video demonstration. https://osf.io/rnz62/files/osfstorage/624829a267553801c7625812/.

**FIGURE 2:**
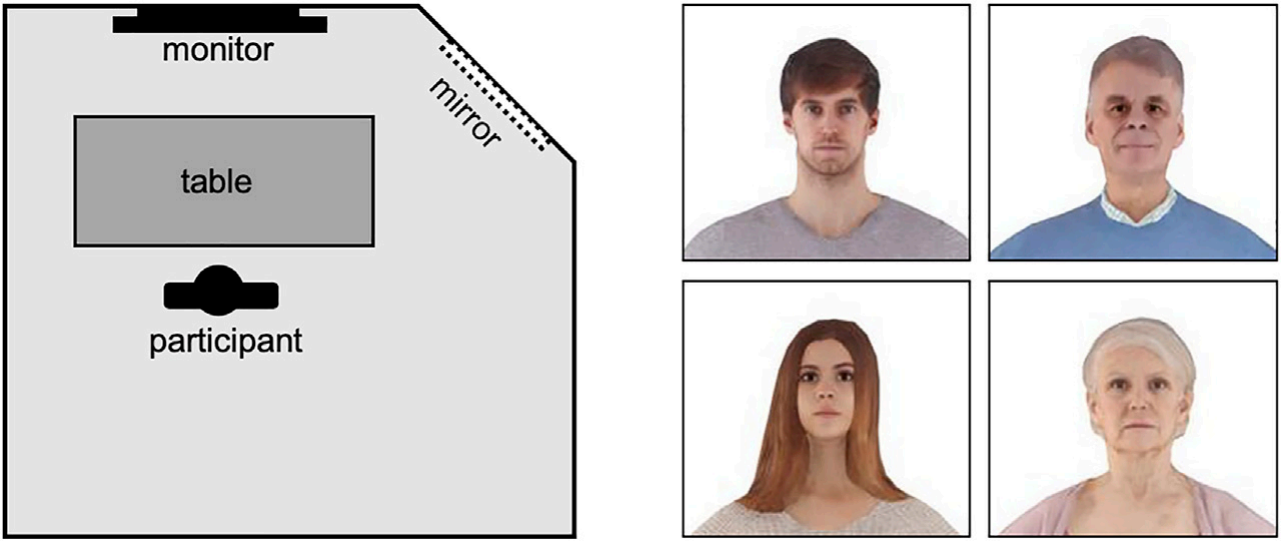
Layout of VR laboratory (not to scale) and avatar faces.

Our studies offered realistic embodiment experiences with virtual avatars of different ages. We created a detailed virtual copy of the laboratory (see Figure 1) that allowed visual, spatial, and tactile orientation. To intensify the body ownership illusion, subjects saw their virtual body in a mirror and obtained direct visual feedback of movement (see Figure 1). A set of four avatars (younger woman, older woman, younger man, and older man) was obtained (see http://www.renderpeople.com/) for the participants’ virtual appearance (Figure 2), as implementing individual character-created avatars exceeded the project’s technical and economical limits. Participants saw their virtual same-gender avatar either as younger or older. For the different participant age groups, the experimental groups consisted of age-incongruent avatars and the respective control group of an age-congruent avatar selection. Thus, participants belonged to either the younger avatar group (YA) or the older avatar group (OA). For readability purposes throughout the manuscript, the YA and OA group abbreviations will be combined with an index letter indicating the subjects’ chronological age group, resulting in the OA_y_ (experimental group) and YA_y_ (control group) identifiers for Studies 1 and 2, representing younger and older age avatars with young participants; furthermore, the group identifiers follow the same setup with older participants in Study 3, with the YA_o_ (experimental group) and OA_o_ (control group) identifiers.

We conducted three independent studies with similar procedures but different age samples and different dependent variables. Study 1 included physical performance measures and Study 2 cognitive assessments. Both Studies 1 and 2 were conducted with younger participants. Study 3 included an older sample that was assessed on both physical and cognitive performance.

The paradigm combined the realism of a field environment with the controllability and standardization and controllability known, for instance, from experimental lab research on the Proteus effect. Our goal was to gain some fundamental knowledge of the valid, immersive, and self-relevant activation of AGS in a VR setting, to capture the increase or decrease in relevant performance parameters, and thus to enable future developments of interventional techniques for the above-mentioned negative effects on health.

Data assessment for the studies was conducted in a time window of several weeks, which was limited by laboratory capacities, the availability of assistance staff, and unforeseen lockdown restrictions due to the COVID-19 pandemic. This particularly affected recruitment for Studies 1 and 2. Within these boundaries, we aimed to reach the determined minimum sample size and meanwhile make maximum use of the resources allocated to the project.

### Hypotheses

Based on previous findings and the characteristics of our design and methods, we present three hypotheses concerning the age avatar embodiments’ effects on the subsamples’ performance scores and their associated moderating parameters.

#### Hypothesis 1

Studies 1 and 2 were expected to provide evidence of the immersive virtual age embodiment and thus activate self-reflexive AS. For the OA_y_ group, in contrast to the YA_y_ group, we hypothesized that our experimental manipulation would result in a cognitive and physical performance decline commonly associated with old age-related developments.

#### Hypothesis 2

After providing evidence of this “virtual aging” effect in a young sample, insights from Study 3 served to provide cross-validation evidence of the reverse direction of our manipulation: the virtual “youth fountain” that makes older people feel and act younger. Even if our virtual old age avatar embodiment were able to activate relevant self-reflexive expectations of the younger adults’ own aging process, the technique would not necessarily be easily reversible. Providing evidence of successful manipulation in both directions would prove our intervention equally applicable to both age groups. Thus, we hypothesized a performance increase for the older sample in the YA_O_ condition, compared to the OA_O_ condition.

#### Hypothesis 3

The design controlled for important covariates and allowed exploration of the possible moderating effects of negative AGS in the experimental group based on previous findings (Kornadt et al., 2016). Because this moderating effect seems particularly strong when the stereotype content is matched with the domain investigated (Levy and Leifheit-Limson, 2009), we considered both domain-general and domain-matched AGS in the analyses. We hypothesized an interaction effect of the domain-general and domain-matched explicit AGS with our experimental intervention on the relevant performance parameters. Participants within the experimental age-incongruent subgroups of all studies were expected to show a stronger age embodiment effect when they carry particularly negative stereotypes of old age and particularly positive ones of young age. Hence, applying a successful VR age avatar embodiment to an older sample and comparing the quantity and quality of results was expected to possibly give insight into the relevant mechanisms responsible for the effect within the subsamples and thus provides evidence of the applicability of our manipulation to older samples concerning possible positive effects on the short- and long-term correlates of the aging process.

### Power Analysis

A priori power analysis using G*Power (Faul et al., 2007) was conducted to determine the sample size necessary. Parameters for the calculations were obtained from the experimental design of the study by Reinhard et al. (2020), which was closest to the current methodological approach and where a comparison of two avatar groups’ walking speed scores before and after applying avatar embodiment led to a Cohen’s *f* of 0.43. The power analysis included a repeated measures analysis of variance (ANOVA) with three measures in total for two groups to compare: an error rate of *α* = 0.05, a power level of *1—β* = 0.95, and a correlation of *r* = 0.50 between measures, and it resulted in a minimum total sample size of *N* = 50.

### Data Analysis

To account for adequate randomization effects concerning possible parameters influencing the main results, the data analysis of each study started with a *t*-test comparison of the two experimental conditions based on relevant baseline parameters: immersion intensity, performance domain-related baseline measure (physical fitness or memory self-efficacy), positive and negative affect, and implicit and explicit AGS. The main analysis for each study was carried out using ANOVA and repeated-measures ANOVA to compare the experimental conditions (with and without age manipulation in VR) concerning their physical performance and cognitive performance accordingly. The statistical assumptions for the inferential tests were checked prior to the analysis and did fulfill the criteria necessary in accordance with the relevant literature (Kaur and Kumar, 2015).

To provide evidence of the robustness of our results against alternative explanations and possible undisclosed associations and to provide evidence of the validity of our approach, an ANCOVA analysis was carried out including the aforementioned moderators and controls as covariates and looking at 1) the main effects of this covariate on performance, 2) the interaction effects with our experimental condition on performance, and 3) whether the ANOVA’s main effect results remained identical.

## Study 1

### Participants

In total, *N* = 68 participants (54 female) aged 18–35 (*M* = 22.46, *SD* = 3.10) were recruited using both online and offline university blackboards and randomly assigned to the experimental (*n* = 35) or control group (*n* = 33). There were more female subjects in the total sample and an almost equal distribution of gender (79 *vs*. 80% female) and age (*M =* 22.12, *SD* = 2.27 *vs*. *M* = 22.77, *SD* = 3.71) in both the control and experimental conditions.

### Procedure

**FIGURE 3:**
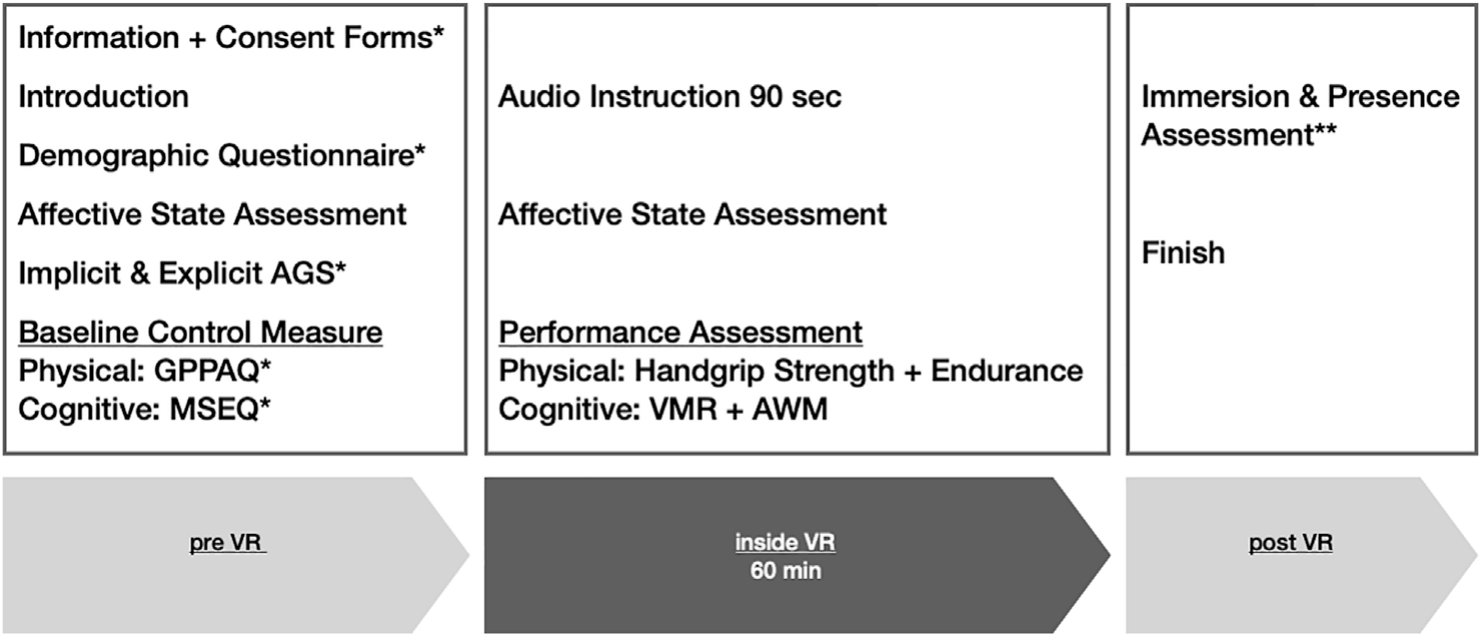
General procedure. Note. Studies 1 and 2 consisted of either the physical or the cognitive performance focus, containing only the baseline and performance assessments of one performance domain as mentioned above. Study 3 included both performance domains into the procedure. * For Study 3 some questionnaires were completed in a paper-pencil setting prior to the laboratory appointment. ** The immersion and presence assessment was only carried out in Studies 1 and 2.

The 75-min procedure (see Figure 3) started with information and consent forms, followed by a demographic questionnaire, an assessment of affective state, measures for implicit and explicit AGS, and a physical fitness questionnaire. The VR headset was then mounted, and motion trackers inside the headset and hand controllers allowed synchronization of the participants’ movements. The avatar conditions’ random assignment was based on a randomization list and was not announced or discussed by the laboratory members. The young participants were assigned to the younger (YA_y_) or older (OA_y_) age avatar group. Participants were presented with a 90-s audio instruction directing their attention to their avatar’s appearance and movements to facilitate adequate immersion. After this introduction, a repeated measure of the affective state was followed by physical performance measures for handgrip strength and endurance. After leaving the 60-min VR scenario, participants completed a presence and body ownership questionnaire.

### Measures

#### Handgrip Strength

The participants’ handgrip strength was measured three times in a row while the VR scenario was running. A handgrip dynamometer was placed into the participant’s dominant hand and no visual reference was presented within the VR. As suggested by Innes (1999), each participant was asked to hold the device with their arm in a neutral position, their shoulder adducted and in a neutral rotation, and their elbow flexed at a 90° angle. The repeated-measures approach was chosen first to obtain several data points for a validity comparison with earlier findings. The observed within-subject effect of declining handgrip scores, *F* (2, 122) = 15.44, *p* < 0.001, *η* ^*2*^ = 0.20, was in line with an expected decline in arm and hand strength from earlier applications (Innes, 1999). Second, the repeated measures approach allowed the assessment of the influence of both initial motivational drive and gradually advancing motivational parameters.

#### Endurance

To measure the participants’ weight-holding endurance, they were instructed to hold the VR controller for as long as possible in a suspended position directly in front of their body with a straight arm and with a 90° angle between the arm and torso. The test ended when the hand was lowered beneath a point at the lower edge of the sternum, which was previously marked with a visual reference outside of VR and thus only visible to the person administrating the test.

#### Implicit Age Group Stereotypes

The Implicit Association Test, which was presented on the VR-integrated virtual monitor, aimed to detected subconscious associations between different mental representations. The general procedure started with learning and practicing a first allocation of positive and negative words in response to certain stimulus categories, including, in this case, age groups and, in other studies, ethnicities (Saujani, 2002) or gender groups (Ramos et al., 2016). Participants were then asked to respond to a reversed allocation of the verbal labels. The resulting “improved D-score,” described by Greenwald et al. (1998), was calculated by including the reaction time difference between the earlier and later phases of the experiment, thus concluding a stronger link of the relevant concept to either positive or negative associations. The task was adapted to AGS and included images of older and younger people together with positive and negative words (e.g., “happiness,” “cruelty”). A negative score represents a stronger association of “old” with negative content and “young” with positive content. To minimize possible priming effects of this presentation, the word list was balanced with positive and negative words and presented in a randomized order.

#### Explicit Age Group Stereotypes

This rating task (Kornadt et al., 2016) consisted of 48 adjectives, equally distributed into positive and negative words and furthermore balanced concerning the factors’ competence and warmth, following the stereotype content model by Fiske et al. (2002). Participants were asked to rate on an 8-point semantic differential scale whether each adjective presented on the virtual monitor applied more to young or older adults. Two separate sum scores were calculated for the positive and negative groups. The positive score was subtracted from the negative one to create an overall AGS score. High values indicate a stronger old AGS negativity, with a strong association of younger age with positive attributes and older age with negative attributes. Lower values indicate fewer AGS with a lesser tendency to view younger people as positive and older people as negative.

A sub-score was calculated, including only the adjectives that were directly related to our dependent construct. The physical AGS score included the positive adjectives “healthy,” “energetic,” “lively,” and “agile” and negative reverse-scored adjectives included “sick,” “lazy,” “powerless,” and “frail.” The resulting score indicated the domain-matched AGS for physical performance.

#### Affective State

Prior to and right after mounting the VR headset, we assessed the participants’ affective state through an adaptation of the Implicit Positive and Negative Affect Test (Quirin et al., 2009) that was presented on the lab monitor outside (but visually alike) the VR scenario. In this projective test setup, participants were asked to rate how much the sounds of. three fictitious words (“BELNI,” “VIKES,” and “TALEP”) match three adjectives describing positive emotions and three adjectives describing negative emotions using a 4-point Likert scale. The authors of the paradigm argue that the obligatory rating of neutral words using positive and negative emotions provides an implicit projection of the participants’ current mood, so no explicit questionnaire is necessary. Positivity and negativity scores before and after mounting the VR headset were calculated as the means of each set of three positive/negative adjectives. The inclusion of affective state development in the analysis aimed to control statistically for possible mood changes after entering the VR scenario, while minimizing social desirability effects by using an implicit assessment. For the following analyses, two mean delta scores were included as moderators, indicating an increase or decrease either in positive or in negative affect during the introduction to the VR scenario.

#### Immersion and Presence

As previous studies have shown VR embodiment effects to be sensitive to variations in the intensity of the immersive experience and presence perception, we used the Igroup Presence Questionnaire (IPQ; Schubert, 2003) and, for presence, the Body Ownership Questionnaire (BOQ; adaption by Reinhardt et al., 2020) in the laboratory monitor to assess the participants’ immersion. The four resulting IPQ sub-scales “spatial presence,” “involvement,” “realness,” and “global presence” were summarized into one total IPQ presence score that was included in the analysis parallel to the body ownership score from the BOQ.

#### Physical Fitness Assessment

Physical fitness was assessed as a baseline control measure prior to the physical performance testing in VR and carried out using the General Practice Physical Activity Questionnaire (GPPAQ; Ahmad et al., 2015). The amount and intensity of physical activity in professional and recreational contexts is assessed and summarized as a total score.

### Results

#### Baseline Group Equivalence

There were no baseline group differences for immersion, *t* (62) = –1.17, *p* = 0.65, presence, *t* (62) = –0.61, *p* = 0.68, physical fitness, *t* (56) = –0.14, *p* = 0.90, positive affect, *t* (63) = 0.20, *p* = 0.85, negative affect, *t* (62) = –0.29, *p* = 0.77, implicit AGS, *t* (57) = 0.67, *p* = 0.49, explicit AGS, *t* (59) = 1.32, *p* = 0.19, and physical performance-matched explicit AGS, *t* (59) = 0.62, *p* = 0.54.

#### Immersion and Presence

The total scores for immersion indicate an equally sufficient level of immersion (*M =* 4.12, *SD* = 0.89) and presence (*M =* 2.27, *SD* = 0.88).

#### Effects of the Experimental Age Manipulation

**FIGURE 4:**
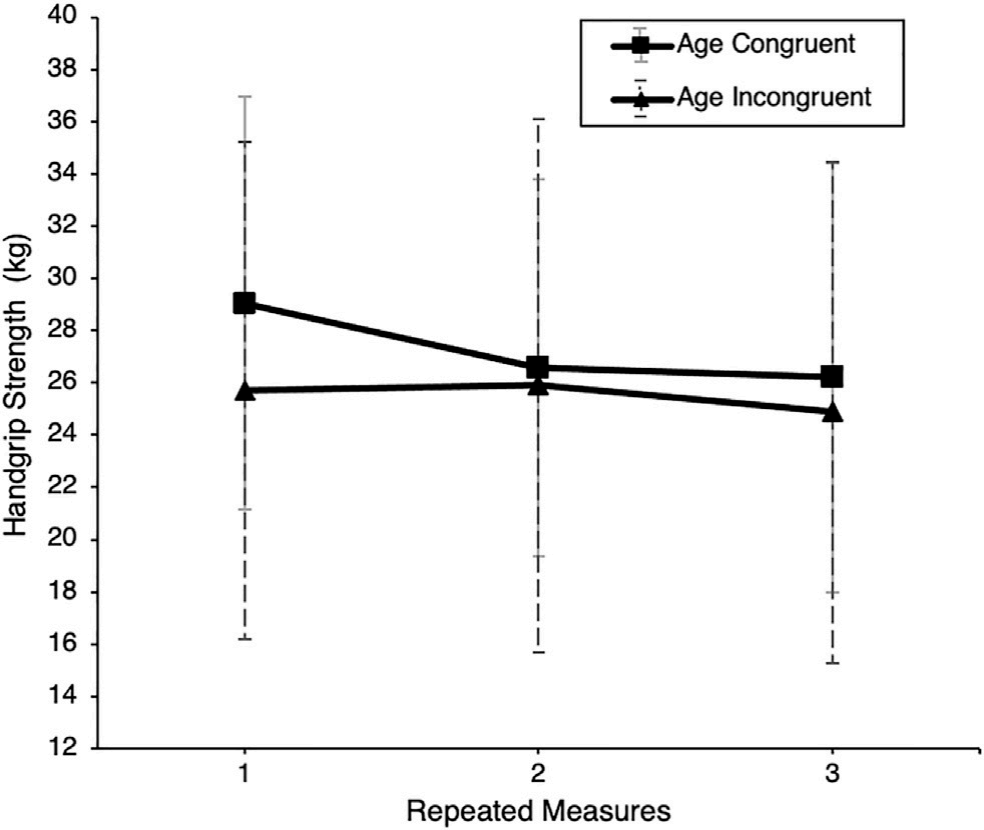
Handgrip strength by experimental condition. Note. Handgrip-strength scores for young sample in kg of YA_y_ (young avatar) and OA_y_ (older avatar) subgroups over three repeated measures.

There was no main effect of the experimental condition on endurance, *F* (1, 60) = 1.52, *p* = 0.22, *η* ^*2*^ = 0.025), indicating avatar age did not affect performance. There was also no main effect on handgrip strength, *F* (1, 61) = 0.71, *p* = 0.40, *η* ^*2*^ = 0.01. However, an interaction effect between avatar age and measurement repetition was found, *F* (2, 122) = 7.06, *p* = 0.01, *η* ^*2*^ = 0.10, indicating a difference in slopes for the two conditions (Figure 4). On closer inspection, this interaction effect is mainly driven by a first measurement in the control group that began exceptionally high and dissolved for the following two measurements, indicating an initial intercept in performance of handgrip or forearm physiology.

#### Controlling for Physical Fitness

There was no main effect of physical fitness on handgrip strength, *F* (1, 54) = 0.45, *p* = 0.50, *η* ^*2*^ = 0.01, or endurance, *F* (1, 51) = 0.28, *p* = 0.60, *η* ^*2*^ = 0.01, and no interaction effect of avatar age with physical fitness on handgrip strength, *F* (1, 110) = 0.02, *p* = 0.90, *η* ^*2*^ = 0.00, or on endurance, *F* (1, 55) = 1.25, *p* = 0.27, *η* ^*2*^ = 0.02. In line with our initial results, no main effect of avatar age on handgrip strength was found, *F* (1, 55) = 0.00, *p* = 0.99, *η* ^*2*^ = 0.00. When controlling for physical fitness, the interaction effect between avatar age and measure repetition remained significant, *F* (2, 110) = 0.41, *p* = 0.02, *η* ^*2*^ = 0.07.

#### Controlling for Affective State

Including the delta scores for positive mood changes during the VR manipulation as a within-subject factor in a repeated measures ANOVA of handgrip strength aimed to control for mood changes that might have strained our results. The analysis revealed no main effect of positive mood change, *F* (1, 56) = 0.63, *p* = 0.43, *η* ^*2*^ = 0.01, and no interaction effect with avatar age, *F* (1, 56) = 3.90, *p* = 0.052, *η* ^*2*^ = 0.07. Compared to our initial findings, the main effect of avatar age on handgrip strength remained non-significant, *F* (1, 56) = 0.08, *p* = 0.79, *η* ^*2*^ = 0.00, and the interaction effect between avatar age and measure repetition remained significant, *F* (2, 112) = 4.10, *p* = 0.02, *η* ^*2*^ = 0.07.

An exploration of negative mood changes as a within-subject factor also revealed no main effect of negative mood change, *F* (1, 55) = 0.13, *p* = 0.72, *η* ^*2*^ = 0.00, and no interaction effect with avatar age on handgrip strength, *F* (1, 55) = 2.52, *p* = 0.12, *η* ^*2*^ = 0.04. In line with our preliminary findings, there was no main effect of avatar age on handgrip strength, *F* (1, 55) = 0.70, *p* = 0.41, *η* ^*2*^ = 0.01, and the interaction effect between avatar age and measure repetition remained significant, *F* (2, 110) = 3.85, *p* = 0.02, *η* ^*2*^ = 0.11. In summary, the results of the avatar age manipulation cannot be explained by changes in the subjects’ affective state.

#### Influence of Age Group Stereotypes

Advancing the analysis of AGS scores as a possible moderator revealed no main effect of AGS on handgrip strength on an implicit, *F* (1, 55) = 0.68, *p* = 0.41, *η* ^*2*^ = 0.01, or explicit level, *F* (1, 52) = 1.31, *p* = 0.26, *η* ^*2*^ = 0.03, and no interaction effect of avatar age with AGS on handgrip strength on an implicit, *F* (1, 52) = 2.01, *p* = 0.16, *η* ^*2*^ = 0.04, or explicit level, *F* (1, 52) = 1.34, *p* = 0.25, *η* ^*2*^ = 0.03. The main effect of avatar age remained non-significant both for implicit, *F* (1, 52) = 2.44, *p* = 0.12, *η* ^*2*^ = 0.04, and explicit AGS scores *F* (1, 52) = 0.16, *p* = 0.70, *η* ^*2*^ = 0.00. For our second dependent variable, endurance, there were no main effects for implicit, *F* (1, 52) = 0.60, *p* = 0.45, *η* ^*2*^ = 0.02, or explicit AGS, *F* (1, 52) = 0.08, *p* = 0.78, *η* ^*2*^ = 0.00.

**FIGURE 5:**
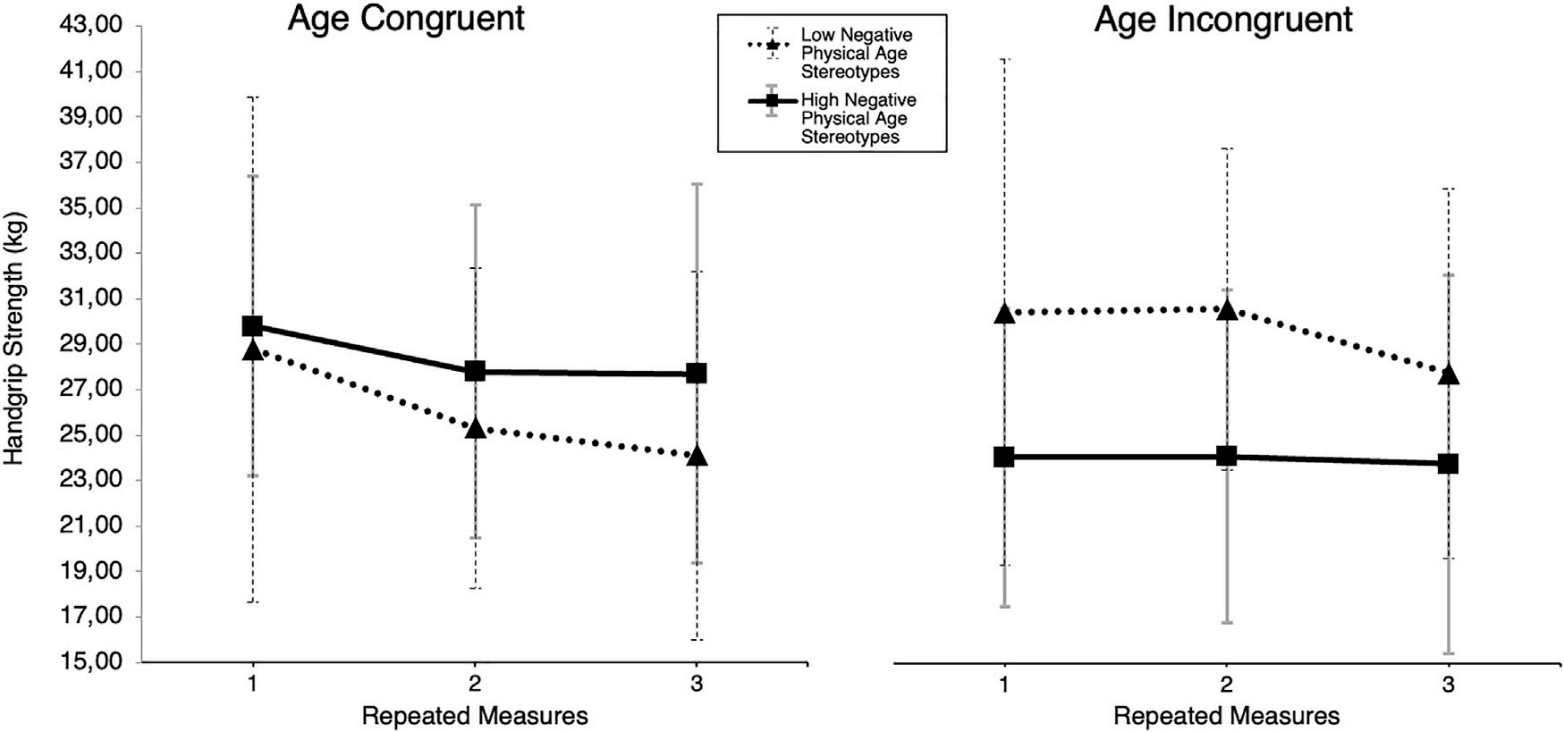
Handgrip strength by experimental condition and physical age group stereotype negativity. Note. Handgrip strength scores of young sample displayed separately for the YA_y_ (young avatar) and OA_y_ (old avatar) subgroups over three repeated measures with separate graphs for (median split) low and high negative domain-matched (physical) AGS.

A separate analysis of domain-matched AGS for physical performance was carried out based on the stereotype-matching effect reported by Levy and Leifheit-Limson (2009). This included a physical performance-related AGS negativity score as a possible moderator of handgrip strength and did not reveal a significant effect of domain-matched AGS on handgrip strength, *F* (1, 52) = 2.0, *p* = 0.16, *η* ^*2*^ = 0.04. A significant interaction effect with avatar age was discovered, *F* (1, 52) = 4.34, *p* = 0.04, *η* ^*2*^ = 0.08, indicating that for the experimental group, only subjects with higher representations of physical AGS performed worse in the handgrip strength task (Figure 5). Only the analysis of lexical subgroups related to physical performance showed a moderating effect in the physical performance domain, which hints at the possible domain-matched relevance of AGS to performance in a selected domain.

### Discussion

The virtual age embodiment did not directly affect handgrip strength. The significant interaction between condition and the repeated measurement, however, indicates that physical performance was affected in a more complex and subtle way than hypothesized. Thus, our first hypothesis is only partially confirmed. Compared to both the pattern shown in the control group and that known from other research (Innes, 1999), participants in the experimental group exhibited a conspicuously low performance in the first measurement; consequently, there was almost no fatigue-related decline in performance, typically found after a major effort. Experiencing being older appears to have resulted in a strategic attempt to save efforts and thus avoid premature resource depletion. This interpretation would be consistent with notions of selective optimization with compensation (Freund and Baltes, 1998) and reminiscent of an anticipated loss-based selection vis-à-vis a potentially challenging task or, as Ebner et al. (2006) would say, a goal orientation guided by loss prevention. The drawback of this interpretation is its assumption that younger people have at least tacit knowledge of the developmental regulation strategies of older individuals. A more simplistic explanation would be that participants approached the unknown task more cautiously, considering the possibly excessive demands they stereotypically expected for older people in unknown performance situations. Our data are compatible with both interpretations and invite further exploration of this issue in future research.

An intriguing finding of Study 1 was the significant interaction of the experimental condition with domain-matched (but not domain-general) explicit AGS, suggesting that AGS activation is indeed the effective mechanism behind our findings. In line with our expectations, participants only behaved stereotypically old in the aspects they believed related to old age. This aligns with research by Levy and Leifheit-Limson (2009), who showed stronger effects when the stereotype content matched the outcome performance domain.

Surprisingly, we did not find significant main effects of avatar age on endurance. A more strenuous variant with a heavier weight might have produced clearer effects, as the subjects’ physical resources would then be depleted more strongly. It could also be that the task did not match the content of the self-relevant stereotypes people hold. As our findings from the handgrip strength task suggest, changes in performance seem quite sensitive to the specific domain and hence to the specific content.

## Study 2

### Participants

Study 2 comprised 45 (34 female) participants aged 19–30 years (*M* = 23.04, *SD* = 1.99) that were assigned to the control (*n* = 24) and experimental conditions (*n* = 21).

### Procedure

The design, procedure, and time spent in VR remained almost identical to Study 1, except the dependent variables included cognitive performance measures instead of physical ones (see Figure 3) and the respective baseline control measure was replaced with a memory self-efficacy questionnaire. The young participants again were assigned to the younger (YA_y_) or older (OA_y_) avatar group.

### Measures

#### Verbal Memory and Recall

To assess verbal memory and recall (VMR) abilities, we adapted the word list reproduction task by (CERAD-WL; Morris et al., 1989). Participants were asked to read aloud a set of 15 words presented for 2 s each. After the first sequence, each participant was asked to reproduce the words from memory. The procedure was repeated twice while changing the order of the words. The requirement of learning and recalling words offers both the inspection of a baseline recall ability and of a learning slope within the repetition of the sequence. The test shows strong associations with other established cognitive tasks (Yuspeh et al., 1998), while older samples are known to perform worse on the test (Hankee et al., 2016) and learning slopes appear steeper for younger individuals (Jones et al., 2011).

#### Alphanumeric Working Memory

In the letter–number sequencing subtest from the Wechsler Adult Intelligence Scale (Wechsler, 2008), a series of numbers and letters was read to the participants to measure their alphanumeric working memory capacity (AWM). Subjects were then asked to reproduce the sequence in a predetermined order. The test included 10 sets of three tasks, with a gradually increasing sequence length. The test ended as soon as the participant gave only false answers to a set of three tasks.

#### Sustained Attention

Reproducing the Testing Battery for Attention Performance (Zimmermann and Fimm, 2017) included a series of geometric figures, each displayed for 2 s, followed by a 1-s fixation cross. Figure variations included assorted colors, shapes, and sizes. Target stimuli were defined as a sequence of two figures with an identical shape, regardless of size or color. Participants were asked to respond as quickly and accurately as possible by pressing a button whenever a target stimulus appeared. Performance was measured as accuracy among 36 target stimuli distributed over 300 trials.

#### Memory Self-Efficacy

As a baseline control variable for the dependent measures, the Memory Self-Efficacy Questionnaire by (MSEQ; Berry et al., 1989) assessed the participants’ expectations concerning their memory performance using everyday examples of memorizing grocery shopping lists, telephone numbers, household items, or digits. For each category, the number of items that participants believed they could memorize was coded, and a total memory self-efficacy score for each subject was calculated following the original procedure.

#### Implicit and Explicit Age Group Stereotypes

Implicit and explicit AGS were assessed in an identical manner compared to Study 1. Again, a domain-matched explicit AGS score was calculated, this time matching the cognitive performance domain using a subset of adjectives, namely, “clever,” “learned,” “literate,” and “versed” and the negative reverse-scored adjectives “forgetful” and “senile.” The resulting score indicated the domain-matched AGS for cognitive performance.

### Results

#### Baseline Group Equivalence

There were no baseline group differences in immersion, *t* (42) = 1.19, *p* = 0.24, presence, *t* (42) = 1.02, *p* = 0.86, memory self-efficacy, *t* (40) = 0.38, *p* = 0.71, positive affect, *t* (42) = –0.06, *p* = 0.95, negative affect, *t* (41) = 1.18, *p* = 0.25, implicit AS, *t* (38) = 1.27, *p* = 0.22, and memory performance-matched explicit AGS negativity, *t* (42) = –1.41, *p* = 0.166. The total score for immersion intensity indicates an equally sufficient level of immersion for both the control (*M =* 0.48, *SD* = 0.15) and experimental conditions (*M* = 0.46, *SD* = 0.12). A baseline difference in general explicit AGS was found, *t* (42) = –2.59, *p* = 0.013, indicating higher baseline AGS scores for the OA_y_ than the YA_y_ group.

#### Immersion and Presence

The total scores for immersion again indicate equally sufficient levels of immersion (*M =* 1.97, *SD* = 0.76) and presence (*M =* 3.64, *SD* = 0.97).

#### Effects of the Experimental Age Manipulation

**FIGURE 6:**
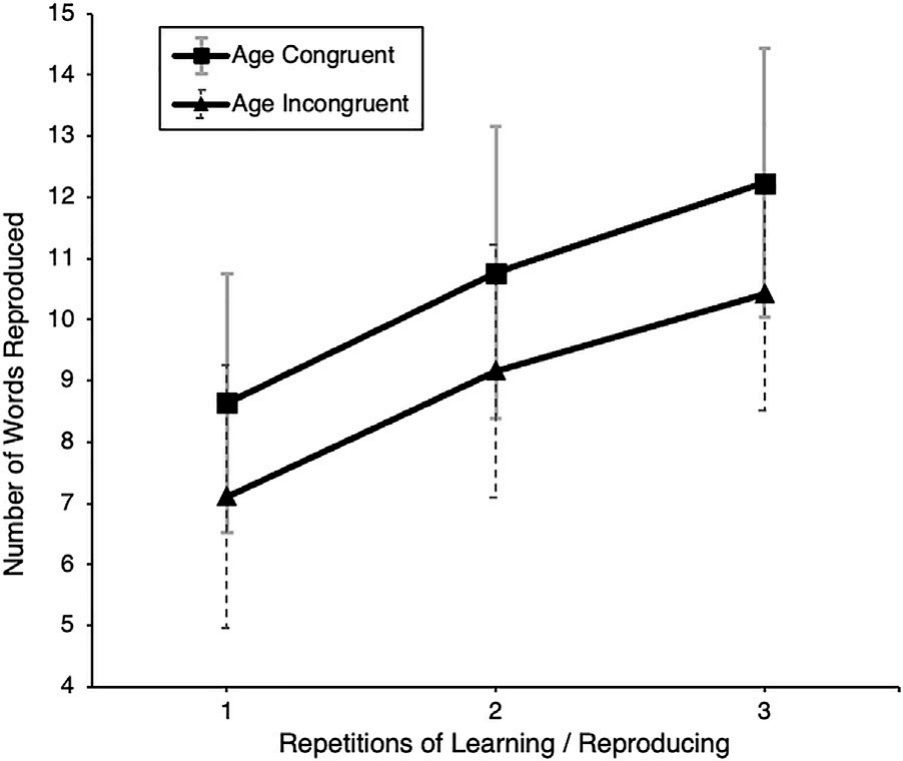
Memory performance (VMR) by experimental condition. Note. Number of reproduced words from the verbal encoding and recall task (VMR) for young sample, displayed separately for the YA_y_ (young avatar) and OA_y_ (old avatar) subgroups over three repetitions of learning and reproducing.

A between-subject comparison of the experimental (OA_y_) and control (YA_y_) groups over three measures of VMR showed a main effect of avatar age, *F* (1, 43) = 8.76, *p* = 0.005, *η* ^*2*^ = 0.17. Participants embodying an older avatar performed worse than those in the control condition (see Figure 6). Further analysis revealed no main effect of avatar age on AWM, *F* (1, 43) = 0.30, *p* = 0.59, *η* ^*2*^ = 0.01, or sustained attention, *F* (1, 40) = 0.006, *p* = 0.94, *η* ^*2*^ = 0.00.

#### Influence of Memory Self-Efficacy

Self-reported memory self-efficacy showed no main effect on VMR, *F* (9, 25) = 0.96, *p* = 0.49, *η* ^*2*^ = 0.26, and no interaction effect with avatar age on VMR, *F* (6, 25) = 0.40, *p* = 0.87, *η* ^*2*^ = 0.09, with the main effect of avatar age moving into the non-significant range, *F* (1, 25) = 3.14, *p* = 0.09, *η* ^*2*^ = 0.11.

#### Controlling for Affective State

There was no main effect of delta scores for positive mood, *F* (1, 37) = 0.08, *p* = 0.78, *η* ^*2*^ = 0.00, or negative mood, *F* (1, 37) = 0.52, *p* = 0.47, *η* ^*2*^ = 0.01, on VMR, while the main effect of avatar age remained significant, *F* (1, 37) = 7.11, *p* = 0.01, *η* ^*2*^ = 0.16. This indicates that changes in the subjects’ affective state during our manipulation likely did not account for the main effects on the dependent variables.

#### Influence of Age Group Stereotypes

The AGS scores were included in separate analyses as a possible moderator. There was no main effect of explicit, *F* (1, 40) *=* 0.06*, p =* 0.80, *η* ^*2*^ = 0.00, or implicit AGS, *F* (1, 36) *=* 1.3*, p =* 0.26, *η* ^*2*^ = 0.04*,* on VMR. In addition, there were no moderating effects of avatar age on the dependent measure for explicit, *F* (1, 40) *=* 0.07*, p =* 0.79, *η* ^*2*^ = 0.002*,* or implicit AGS, *F* (1, 36) *=* 0.77*, p =* 0.39, *η* ^*2*^ = 0.02, while the main effect of avatar age on VMR remained significant with the inclusion of explicit, *F* (1, 40) *=* 5.15*, p =* 0.03*, η* ^*2*^ = 0.12, and implicit AGS, *F* (1, 36) = 6.67*, p =* 0.01, *η* ^*2*^ = 0.16.

**FIGURE 7:**
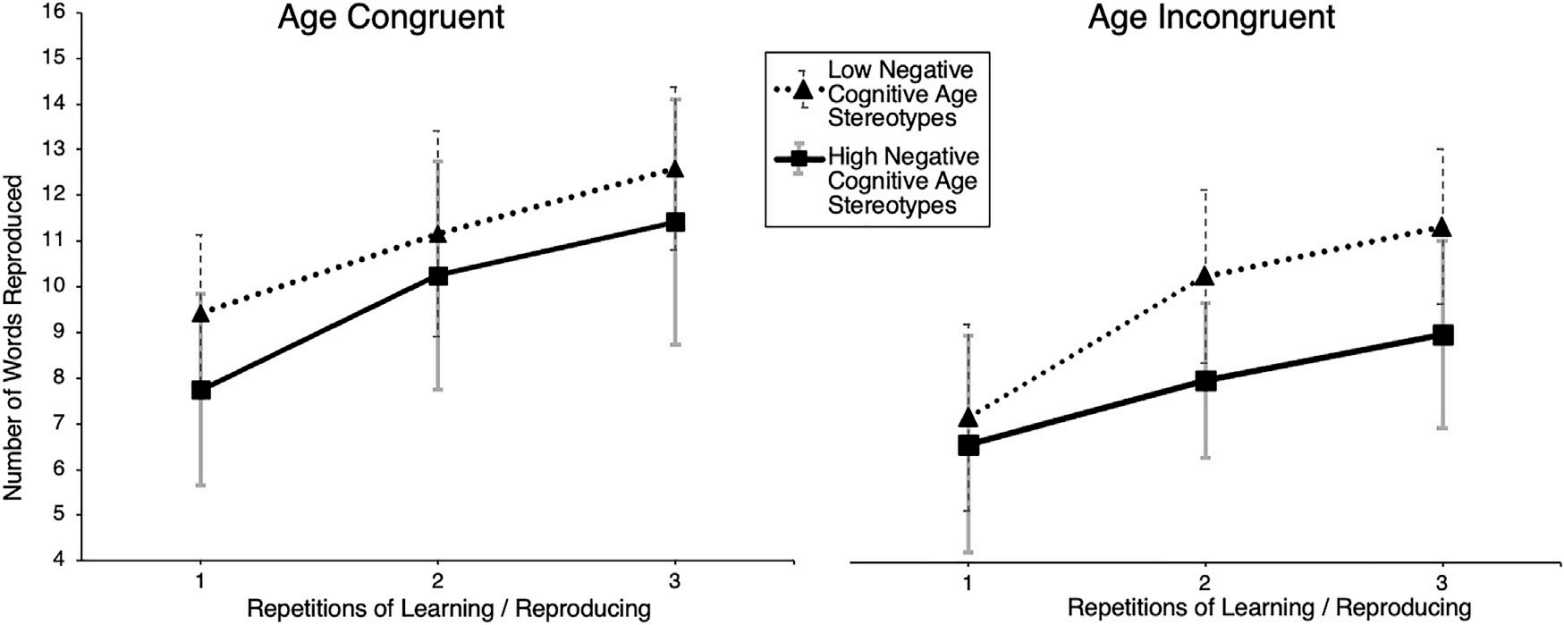
Memory performance by experimental condition and cognitive age group stereotype negativity. Note. Number of words reproduced from the verbal encoding and recall task of young participants, displayed separately for the YA_y_ (young avatar) and OA_y_ (old avatar) subgroups over three repeated measures with separate graphs for (median split) low and high negative domain-matched (cognitive) AGS.

A domain-matched operationalization of explicit AGS was included as a between-subject factor, similar to in Study 1 but now for cognitive performance, revealing no main effect of cognitive performance-related AGS on VMR, *F* (1, 40) *=* 0.02, *p =* 0.90, *η* ^*2*^ = 0.02*.* No interaction effect of the repeated measures with cognitive performance-related AGS was found, *F* (1, 40) *=* 0.12*, p =* 0.73, *η* ^*2*^ = 0.003*.* Due to the small sample size, we desisted from separate statistical analyses for each measurement and instead performed a descriptive inspection of the data. Here, participants from the experimental group who represented higher cognitive AGS scores showed a notably lower score in the first and second of the three consecutive measurements (see Figure 7).

### Discussion

In Study 2, a multifaceted selection of cognitive tasks was used. Even though the required sample size was not reached and the study was possibly underpowered, we found a strong main effect of avatar age on VMR. With strong connections to the participants’ motivation and persistence, this finding is in line with our expectations, and the effect size of *η* ^*2*^ = 0.18 is large. For Study 2, the first hypothesis was confirmed. However, not all cognitive measures applied in this study showed a meaningful difference concerning avatar age condition. Most importantly, it is unclear why this experimental approach affects cognitive performance more strongly than physical performance. A certainly obvious difference lies within the details of test application. The cognitive measure of VMR was carried out right inside the virtual scenario with the relevant words displayed on the virtual monitor. Subjects were forced to direct their attention to the general direction of the monitor and mirror. Meanwhile AWM and handgrip strength tests were both administered without any visual reference within the VR scenario. Perhaps stereotypes related to cognitive performance decline in old age are more widespread than those targeting physical performance (Levy, 2003). However, it must be noted that the main effect of avatar age on VMR was no longer significant when the implicit AGS score was included as a covariate. Unfortunately, a post-hoc inspection of the correlations between implicit AGS and the experimental condition, as well as memory performance did not provide a clear picture to explain this finding. More research is needed to understand the role of implicit AGS in this context.

Interesting results were also obtained when including domain-matched explicit AGS as a moderator in the analyses. We found a substantial offset in the learning slopes in the experimental older avatar group. A descriptive inspection of the data could indicate that strong domain-matched negative AGS were associated with low performance expectations for the older avatar group prior to the experiment, leading to a steeper learning curve once the task started and once participants gained confidence. Examining the data more closely, however, leads to another possible interpretation. The largest difference in performance occurred at the first measurement, where participants differing in their domain-matched AGS also showed a considerable offset in their performance. This finding resembles the handgrip strength effect from Study 1, and it might indicate the use of strategies to prevent the depletion of cognitive resources by using limited resources economically.

Meanwhile, the validity of these findings stands in question, as a significant baseline difference in this moderator was found between the experimental conditions, and future research would require higher sample sizes with careful randomization or matching of participants.

The effect we found for VMR, as well as its large size, cannot be generalized across all cognitive tasks. Data from the AWM task did not show significant effects following avatar age manipulation. One possible interpretation considers the limited capacity of people’s working memory (Baddeley & Hitch, 1974). With limited resources on a neuro-functional level, variations in self-relevant AGS activation and motivational aspects may not account for these performance differences.

## Study 3

### Participants

Study 3 comprised *N* = 117 (66 female) participants aged 50–83 years (*M* = 61.23, *SD* = 7.5) that were assigned to the control (*n* = 59) and experimental conditions (*n* = 58). The recruitment for Study 3 resulted in a much larger sample size compared to the earlier studies, whose recruitment was considerably limited by, for instance, the availability of student participants during certain times in the semester. An age range starting at 50 was selected for two reasons. First, other studies on aging and on AGS sometimes (e.g., Wurm et al., 2007, 2010) include participants who do *not yet* fall into the category of being old to include this transition phase and to study precursors of aging. To cover both groups in the data analysis, chronological age will be included as a covariate. Second, we were guided by more pragmatic reasons given the demanding recruitment situation. The parents and relatives of the university students were in their 50 s or 60 s and could thus be recruited more easily than the general public during the COVID-19 pandemic.

### Procedure

Recruitment was carried out using a university blackboard, leaflets in a campus-attached GP practice’s waiting room, and newspaper bulletins. Prior to the VR assessment, the participants received a bundle of documents by mail and were asked to complete them prior to visiting the VR lab. This included information and consent forms on the study, together with questionnaires on demographic variables, explicit AGS, and memory self-efficacy that were identical to aforementioned studies but carried out in a paper-pencil mode at home. By transferring these questionnaires to a paper-pencil assessment prior to the laboratory appointment, the procedure for Study 3 was deliberately reduced in comparison to Studies 1 and 2 (see Figure 3). As the earlier procedure had reportedly been demanding on the physical and cognitive levels, a trade off had to be reached with a set of sufficiently valid and reliable performance measures and a minimization of possible fatigue effects toward the end of the laboratory appointment. The resulting 75-min procedure started with the assessment of implicit AGS. Participants were then introduced to the lab room, and the VR headset was mounted. Participants were assigned to the younger (YA_o_) or older (OA_o_) avatar group, and the introduction procedure remained identical to Studies 1 and 2. The following assessment of performance measures included verbal memory and alpha-numerical working memory, followed by the handgrip strength task. After a break of 2 minutes, the arm-holding endurance task was administered.

#### Dependent Measures

The participants’ handgrip strength measurement was identical to Study 1. Due to a systematic operational error by one of the four test conductors, some handgrip measurements had to be excluded from the analysis, resulting in 89 complete datasets for this variable. Weight-holding endurance was assessed identical to Study 2 with an additional 500-g wristband attached to the relevant wrist. The additional weight was expected to reduce the overall testing time and variance in results, which both had been unexpectedly high in Studies 1 and 2. The verbal memory and recall and alphanumeric working memory assessments remained identical to previous studies.

### Results

#### Baseline Group Equivalence

There were no baseline group differences for memory self-efficacy, *t* (115) = –0.88, *p* = 0.80, but a significant baseline group difference in general explicit AGS, *t* (115) = –0.96, *p* = 0.02, was found, indicating stronger explicit AGS for the experimental OA_y_ condition.

#### Effects of Experimental Manipulation on Physical Performance

There was no effect of avatar age group (YA_o_ *vs*. OA_o_) on handgrip strength, *F* (1, 87) = 0.85, *p* = 0.36, *η* ^*2*^ = 0.01. Including chronological age as a covariate revealed no main effect of age, *F* (1, 85) = 2.74, *p* = 0.10, *η* ^*2*^ = 0.03, and no interaction effect of age with avatar age group on handgrip strength, *F* (1, 85) = 2.56, *p* = 0.11, *η* ^*2*^ = 0.03.

**FIGURE 8:**
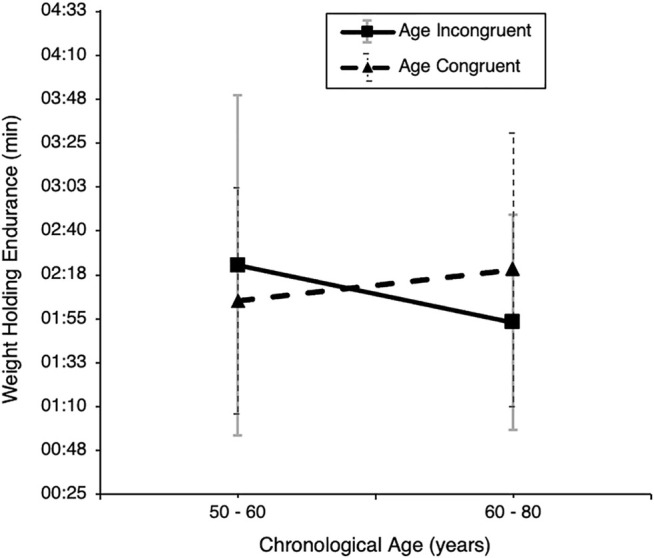
Weight holding endurance of the older sample by experimental condition and median-split chronological age. Note. Weight holding endurance in minutes of older participants, displayed separately for the YA_O_ (young avatar) and OA_O_ (old avatar) subgroups with separate data points for (median split) chronological age.

For weight-holding endurance, there was no main effect of avatar age group, *F* (1, 115) = 0.09, *p* = 0.77, *η* ^*2*^ = 0.00. Further, with chronological age as a covariate, there was no main effect of age, *F* (1, 113) = 0.03, *p* = 0.87, *η* ^*2*^ = 0.00, but a significant interaction effect of chronological age with avatar age group on weight-holding endurance, *F* (1, 113) = 4.54, *p* = 0.035, *η* ^*2*^ = 0.039 (see Figure 8). The 50–60-year-old subgroup of the experimental YA_o_ condition showed a stronger performance than the older 61–83-year-old subgroup of the same avatar condition. For the OA_o_ control condition, the findings were contrary, as the older 61–83-year-old subgroup showed a stronger performance than the younger 50–60-year-old subgroup of the same avatar condition.

#### Effects of Experimental Manipulation on Cognitive Performance

The analysis revealed no main effect of avatar age group on verbal memory and recall, *F* (1, 115) = 0.04, *p* = 0.85, *η* ^*2*^ = 0.00. Further, while a main effect of chronological age on verbal memory and recall was found, *F* (1, 114) = 8.26, *p* = 0.005, *η* ^*2*^ = 0.07, indicating lower scores for the participants with a higher age, no interaction effect of age with avatar age group on verbal memory and recall was found, *F* (1, 113) =0 .18, *p* = 0.68, *η* ^*2*^ = 0.00.

Alphanumeric working memory showed no main effect by avatar age group, *F* (1, 115) = 0.23, *p* = 0.63, *η* ^*2*^ = 0.00. Including chronological age again revealed a main effect, *F* (1, 113) = 9.57, *p* = 0.00, *η* ^*2*^ = 0.08, with lower scores associated with higher age, but there was no interaction effect of age with avatar age group, *F* (1, 113) = 0.43, *p* = 0.51, *η* ^*2*^ = 0.00.

In the absence of significant main effects of our experimental manipulation on the relevant dependent variables, we did not consider any further control or moderator variables.

### Discussion

The results of Study 3 suggest that what we have obtained for the young sample cannot be simply generalized to the older group. Most importantly, we did not find any evidence of the idea that embodying a younger avatar leads to improved performance parameters, so our second hypothesis was not confirmed. Thus, this part of the project was unable to provide the expected cross-validation. Possibly, the most prominent explanation is that the virtual avatars’ incongruence with the subject’s own appearance itself might have caused the observed performance decline due to an increased cognitive load (Sweller, 2011) caused by distraction or excitement. For the age-different avatar in both the young and old samples, this visual incongruence was possibly stronger than for the avatar that was closer to the subject’s own chronological age. For future research, an additional control condition with, for instance, a gender/age-neutral mannequin or a personalized avatar appears a reasonable step to provide better insight into the relevant mechanism of our virtual reality approach. Furthermore, Study 3 was expected to provide evidence of the development of negative AGS interventions aimed at improving long-term health parameters known from previous research and thus minimizing negative effects during the aging process. Such effects were not found, even though the sample size exceeded what was necessary based on our previous power analysis and the selected performance measures were proven reactive to the intervention among younger participants. The roles of several possible influential parameters remain unclear and must be explored in future studies: a strong variation in chronological age within the two age groups was matched with fixed-age avatars, which poses the risk of strong intra-group variations in self-relevant age perceptions compared to the avatar. Interactional findings from Study 3 support this argument, as participants with a chronological age closer to that of the virtual avatar’s age performed better in the endurance task in both the YA_o_ and OA_o_ groups. Again, a baseline difference between experimental conditions in explicit negative AGS was found, which poses a limitation in the results and requires careful consideration in future experimental designs and randomization or matching procedures. Furthermore, the application of our performance tasks might have caused a stereotype threat (e.g., Hess et al., 2003), inducing higher stress among the older sample, thus diluting our results.

## General Discussion

Our research introduced a novel, sustainable, and flexible approach to activating self-relevant AGS and decreasing effects on physical and cognitive performance within VR. It advances the empirical validity of the aging game and outpaces both the experimental effects of priming and the immersive character of false-feedback approaches. It offers sufficient manipulation of the self-relevance of pre-existing AGS with a direct connection to the relevant performance domains and enables the further development of interventional strategies with a hopefully positive impact on various health parameters linked to strong AGS negativity concerning older age groups.

We hypothesized a performance decline among young participants (Study 1 and 2) that embody an older avatar in comparison to a same-aged avatar, which was partially confirmed. We also expected older participants (Study 3) to perform better when embodying a younger avatar in comparison to an older avatar, which was not confirmed. In addition, we hypothesized a general interaction effect of explicit AGS with avatar age embodiment on performance. This hypothesis was only partially confirmed, as the expected association did occur in certain results, but not throughout the three studies. Given the contrastive findings of our intervention for different age groups and different performance parameters, we see several reasons that the hypothesized mechanisms for changing physical and cognitive performance did not affect the older and younger samples alike.

First, the younger and older samples possibly differed concerning their openness to new technology and their earlier experiences with digital technology and VR (e.g., Elias et al., 2012). Individuals who find themselves in a research setup that is considerably unknown to them could experience higher arousal and maybe a stronger decline in cognitive focus or motivation. Unfortunately, the affective state measurement was not applied in Study 3, so we could not test this explanation directly. Even though the immersion scores were sufficient in Studies 1 and 2, the immersion assessment was not applied to the older sample in Study 3, so it cannot be guaranteed that VR scenario immersion was as strong for the older participants, Further, in an explanation that is focused more strongly on resource availability and allocation, one must admit that performance is always tied to the availability of resources, both in the body and the brain, and it is always easier to diminish such resources than to build or activate them only, especially in the short-term setting of a laboratory experiment. For instance, performance will immediately decline after a cognitive overload (Decroix et al., 2016), distraction (Graydon and Eyesenck, 1989), or the activation of affective states (Rader and Hughes, 2005). Vice versa, activating a resource that will then immediately boost performance is obviously much more difficult to achieve. Surely, the activation of self-relevant AGS also does not affect physiological or neurological functioning directly, at least not in the brief period of a lab experiment. Muscles are not weakened or strengthened, and the brain is not slowed or sped up; instead, the utilization of available resources is influenced by the activation of self-relevant AGS. In hindsight, it is not surprising that we found no significant effects for the more basic measures and significant effects for measures with a stronger motivational component.

Furthermore, one must consider group differences in the lifetime in which they were exposed to the self-relevant stereotypes. *Self-reflexive* AGS could be much more difficult to change among older than younger people. Older participants have had much longer exposure to negative old AGS and thus have internalized them more strongly (Levy et al., 2012). Furthermore, the respective old AGS might have become more self-relevant as the individuals have grown older, resulting in a strong and long-lasting, now activated, and highly relevant negative AS. In comparison, the younger participants had much shorter exposure to the negative old AGS held by society; furthermore, these old AGS are not yet self-relevant to them.

Old AGS are acquired early in life (Flamion et al., 2020), are nurtured throughout the lifetime in an environment that tends toward ageism (Naegele et al., 2018), are easily triggered by obvious features (Mason et al., 2006), and function *via* both explicit and implicit pathways (Hess et al., 2003), making them seemingly susceptible to relatively subtle experimental manipulation.

Finally, there is a difference in perspectives of the stereotype content. For young participants, the VR manipulation resembles a “time machine” into a future that they naturally have not yet experienced. Older participants, however, experience a travel into a past they have lived through already. Our VR manipulation for young people might have activated what they associate with old age, while the content is not self-relevant and not strongly represented in their minds. For older participants, we activated both personal knowledge and self-relevant stereotypes about early and late sections of their life span. For this reason alone, our apparently symmetrical study design was probably not symmetrical at all in its psychological implications.

### Strengths and Limitations

Our research proposes a novel approach to studying the short-term effects of activating self-relevant AGS in the lab, and this approach has obvious strengths when compared to more conventional paradigms (Varkey et al., 2006). Our studies, however, also have several weaknesses that might be responsible for the lack of significant findings or might limit the generalizability of our results. The conditions for sample recruitment led to a highly selective sample, as psychology students from our university might have been aware of the content or intention of our study or might have recognized some assessment procedures. The older sample was recruited from a university blackboard, a nearby GP’s waiting room, and newspaper bulletins, which might have caused a somehow selective sample with higher characteristics on positive health behavior, openness to science in general, or interest in VR applications in particular. Such selectivity effects are known from cross-sectional age-comparative studies, even if all relevant measures are taken to prevent them (e.g., Lüdtke et al., 2003). In addition, the sample size was relatively small, especially in Study 2, and our careful randomization procedure nevertheless resulted in partial baseline differences in the experimental conditions concerning the relevant covariate of explicit AGS. A possible lack of identification with the virtual avatar might have resulted from the lack of personalization of the avatars (see Liu et al., 2019). The set of four avatars was chosen to ensure a maximum coverage of our participants’ visual features but, as the avatars were not further individualized, a possible inter-individual variation in avatar identification must be mentioned as a limitation of this piece of research. In addition, more movement trackers could have improved the body illusion effect (Kim et al., 2020). These latter two limitations of the immersion, and hence the validity of our effects, were due to economic and technical factors. In Study 3, several questionnaires were moved from a laboratory assessment to paper-pencil questionnaires prior to the laboratory appointment to make the latter less time-consuming and less exhausting for the older sample. This might limit the comparability of our questionnaire assessments in an age group comparison.

We found that the average endorsement of items measuring self-reported immersion was not the highest possible. Instead of lamenting this fact, we want to draw two other conclusions. First, it seems the VR approach is quite effective in activating self-relevant stereotypes, even when the experience of immersion is not perfect. This is a promising result for future applications of the paradigm. Second, there is room for improvement, and higher immersion could have been obtained with a higher-resolution VR headset, more individualized avatars, sensory feedback on more levels, or a virtual room matching the subjects’ home environments. As stronger effects on cognition and behavior with higher immersion are expected, the full potential of the VR approach is yet to be explored.

Another possible weakness is the possibly invalid outcome variables that were not directly affected by AGS activation. Especially in Study 3, we reduced the overall testing time for pragmatic reasons but meanwhile combined two demanding performance measures in one session. We needed to focus on certain variables and deliberately decided to have the widest possible range of performance domains vis-à-vis the given constraints rather than assessing only one aspect in full. Future research can investigate more diverse outcome variables with the VR paradigm.

It should be noted that concerning the moderating role of pre-existing AGS, they were not manipulated experimentally. Hence, our interpretation that this finding identified the actual mechanism behind the performance declines lacks causal evidence. Future research must manipulate both virtual age *and* the valence of stereotypes to prove the entire causal chain. Furthermore, the moderating role of pre-existing AGS in both handgrip strength and VMR performance appears similar only at first glance. Upon closer inspection, the initial performance is impaired in both tasks, but only for VMR do we find a compensation of this impairment, as indicated by a steeper learning curve. It would be premature to draw substantial conclusions from this difference in performance patterns, as it would generalize this moderating effect too broadly.

One further limitation is that the test administrators were completely aware of both the assignment to one of the two conditions and to the research question, which might have led to a Rosenthal effect (e.g., Dumke, 1978). Their own expectations or AGS might have resulted in differences in behavior toward the control and experimental condition participants. Blinding the test investigators *via* automatic selection of and assignment to the conditions appears a feasible adjustment in the future.

### Conclusion

As Marilyn Ferguson and Toms, (1982) stated, “Of all the self-fulfilling prophecies in our culture, the assumption that aging means decline and poor health is probably the deadliest” (*p*. 272). For this reason, research on self-relevant AGS deserves more attention. At the same time, this research faces the fundamental challenge of manipulating age experimentally. We can therefore come closer to an understanding of the causal processes that link stereotypical attitudes to biological and psychological outcomes. Fortunately, current technology allows us to close the gap between what is possible and what would be needed from a methodological perspective. With the advent of VR applications, we might overcome at least some of the obstacles related to lifespan experimental research.

### Open Practices Statement

The data and materials for all experiments are available at https://osf.io/rnz62/?view_only=d5a447aec74a4aeeab73e8eb38f0a8a1.

All of the experiments were preregistered using OSF-Framework:

1) Physical performance: https://osf.io/dbns9
2) Cognitive performance: https://osf.io/w2msp

## Data Availability

The original contributions presented in the study are included in the article/Supplementary Material, further inquiries can be directed to the corresponding author.

## Author’s Note

Data collection for this paper was conducted from October 2019 to April 2020. Many thanks to Pointreef for their continuous technical support of the VR scenario and to Sebastian Unger for programming the experiments.
